# Efficacy of verum and sham acupoint catgut embedding for treatment of obesity: Study protocol for a randomized controlled trial

**DOI:** 10.1186/s13063-019-3730-8

**Published:** 2019-11-27

**Authors:** Yu-Mei Zhou, Bing Yan, Wei-Qu Yuan, Hai-Bo Yu, Zhuo-Xin Yang

**Affiliations:** The Fourth Clinical Medical College of Guangzhou University of Chinese Medicine, Shenzhen, 518033 GuangDong China

**Keywords:** Acupoint catgut embedding, Obesity, Randomized controlled trial, Visceral fat area

## Abstract

**Background:**

Obesity has become a major public health hazard with epidemic proportions, affecting adults, adolescents, and children of both genders. Previous studies have suggested that acupoint catgut embedding (ACE) might be a potential therapeutic approach for obesity. The purpose of this study is to conduct a rigorous and normative trial to determine the efficacy of ACE for obesity.

**Methods/design:**

A total of 99 eligible patients diagnosed with obesity will be recruited in this study. They will be randomly allocated to either the verum ACE group, sham ACE group, or waiting list (WL) group, with 33 patients in each group. Each patient in the two ACE-based groups will receive eight sessions of treatment, lasting over 8 weeks. The primary outcome is the reduction of body mass index (BMI) after treatment. Secondary outcomes will include waist circumference (WC), hip circumference (HC), waist:hip ratio, body fat percentage, blood lipid level, subcutaneous fat area, visceral fat area, and World Health Organization Quality of Life (WHOQOL). All the outcomes will be evaluated at baseline, at the end of the 8 weeks of treatments, and at 3 months of follow-up. The evaluators and data analyzers will be blinded to group allocation.

**Discussion:**

The findings of this randomized, sham-, and WL-controlled trial will help to investigate the influence of ACE on clinical variables as well as visceral fat area of obesity, which will provide high-quality evidence on the efficacy of ACE for obesity.

**Trial registration:**

Chinese Clinical Trial Registry, ChiCTR1800020248. Registered on December 21, 2018.

## Background

Obesity is an increasing global public health issue characterized by the rise of body fat tissues. Genetic, dietary, lifestyle, and environment factors can all induce obesity. The prevalence of obesity has doubled over the past 10 years [1]. In 2015, in the US, more than 20% of the population was identified to be overweight or obese, being very common in adults [2]. According to Global Health Observatory (GHO) data released in 2016, 39% of adults suffered from overweight or obesity. To date, the prevalence of obesity is increasing in both developed and developing countries. Obesity is associated with increased risk for developing a range of comorbid conditions, such as type 2 diabetes (T2D) [3], cardiovascular disease (CVD) [4], fatty liver disease, and gastrointestinal and psychological disorders [5]. For instance, a number of scholars have reported that obese adults showed a 50% increased risk of developing T2D compared with normal-weight adults [6] and were twice as likely to have hypertension and CVD [7]. Nowadays, obesity and its complications impose a heavy burden on socio-economic development; the World Health Organization (WHO) considers obesity as one of the most serious public health problems worldwide [8, 9].

Methods to treat obesity such as lifestyle modification (specifically dietary modification and regular exercise), surgery, drugs, and complication therapy, are all referred to by the guidelines released by the National Heart, Lung and Blood Institute (NHLBI) of National Institutes of Health (NIH), American College of Cardiology (ACC), etc. [10]. The primary and the most valid recommendations for treating obesity are to restrict the intake of high calorie diets and more physical activities. However, these two methods are often challenging for patients to adopt long-term due to lifestyle and economic issues [11]. Additionally, according to the Cochrane Database of Systematic Reviews, there is no sufficient evidence to indicate that the short-term adjustment of food consumption and physical activity leads to long-term weight loss [12, 13]. On the other hand, anti-obesity drugs seem to be questionable in terms of their efficacy and safety as the adverse events (AEs) associated with these drugs often have negative consequences, such as headache, dizziness, nausea and vomiting, insomnia, etc. The US Food and Drug Administration (FDA) in 2010 [14, 15] recommended against and prohibited the use of some anti-obesity drugs because of serious liver damage and the high risk of CVD. Additionally, rimonabant was found to induce anxiety, depression, and other mental disorders [16]. Consequently, identification of effective and low-risk interventions is highly essential for obese individuals.

Acupoint catgut embedding (ACE), a complementary and alternative therapy, has been used for several decades to treat a variety of disorders, such as perimenopausal syndrome, chronic urticaria, depressive disorder, refractory insomnia, obesity, sciatica, etc. [17]. ACE involves the weekly infixing of surgical chromic catgut sutures into the subcutaneous tissue of the extremities and abdomen with a specialized needle under aseptic conditions. Owing to the continuous acupoint stimulation by the implanted sutures, ACE was considered to be more effective than ordinary acupuncture or electroacupuncture for patients with obesity [18]. Previous research revealed that the body weight and body mass index (BMI) of obese patients could be remarkably decreased after ACE therapy [19]. Moreover, ACE could adjust the imbalance of obesity-related hormones, e.g., leptin, ghrelin, and adiponectin, to reduce body weight [19, 20]. In addition, visceral fat accumulation is often accompanied by obesity, which has been recently reported to play a vital role in the development of metabolic syndrome, a cluster of diabetes, dyslipidemia, and hypertension [21]. A randomized controlled trial (RCT) conducted by Lei et al. [22] revealed that electroacupuncture treatment could reduce BMI and waist circumference (WC), as well as visceral fat area (VFA). However, whether ACE can improve the VFA of obese patients and what the relationship is between VFA and obesity-related indices are still uncertain. Only a limited number of RCTs have investigated VFA changes with regard to ACE treatment for obesity.

Hence, this study is designed as a RCT to assess the effectiveness and safety of ACE for treatment of obesity.

## Methods/design

### Study design

**Fig. 1:**
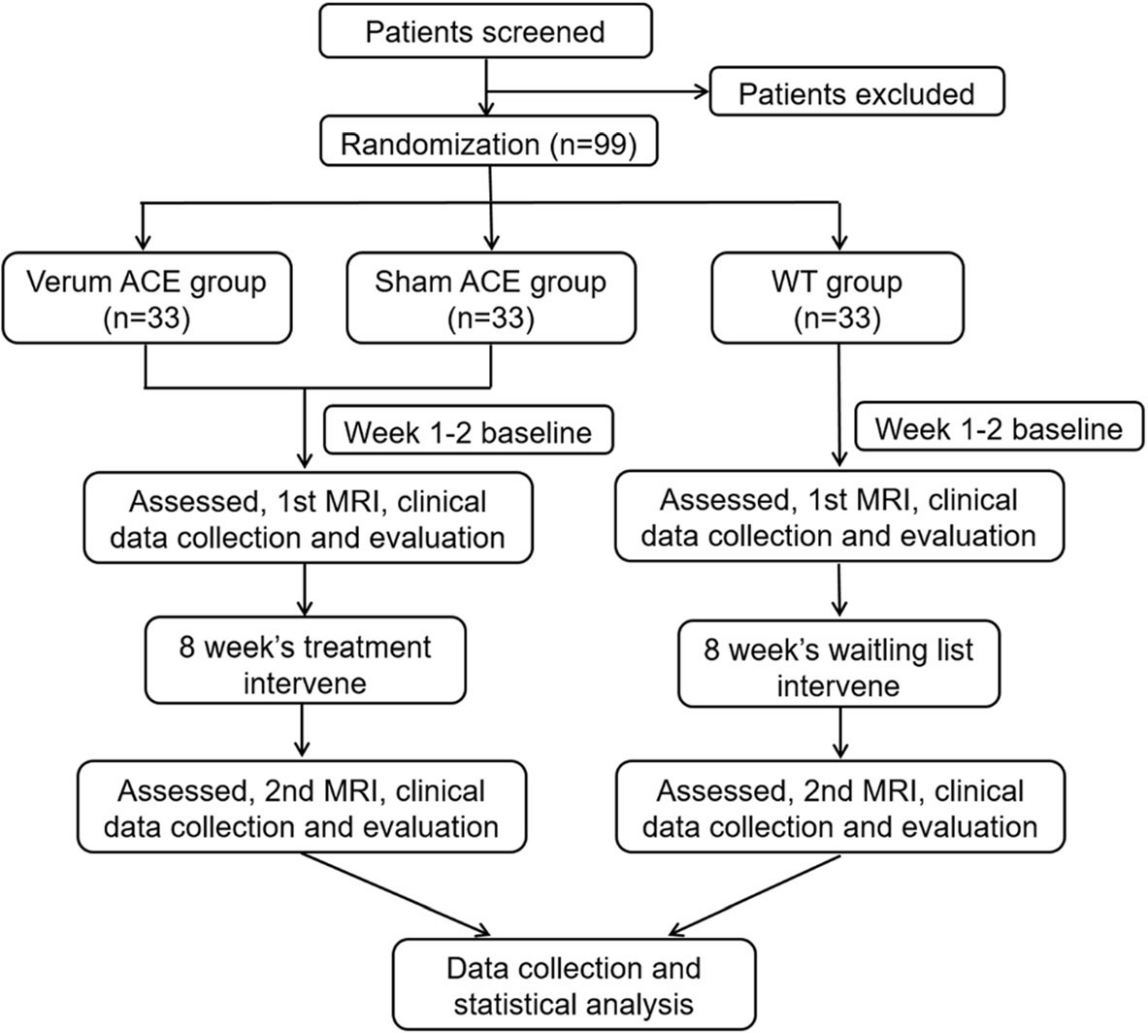
Flowchart of the study design

This single-center, randomized, sham-controlled trial will be conducted at Shenzhen Traditional Chinese Medicine Hospital (Shenzhen, China). The study protocol was approved by the Ethics Committee of Shenzhen Traditional Chinese Medicine Hospital. The study will be conducted in accordance with the 1975 Declaration of Helsinki and the Standard Protocol Items: Recommendations for Interventional Trials 2013 Checklist (Additional file 1). A flowchart of the research procedure is shown in Fig. 1.

### Patient recruitment

**Fig. 2:**
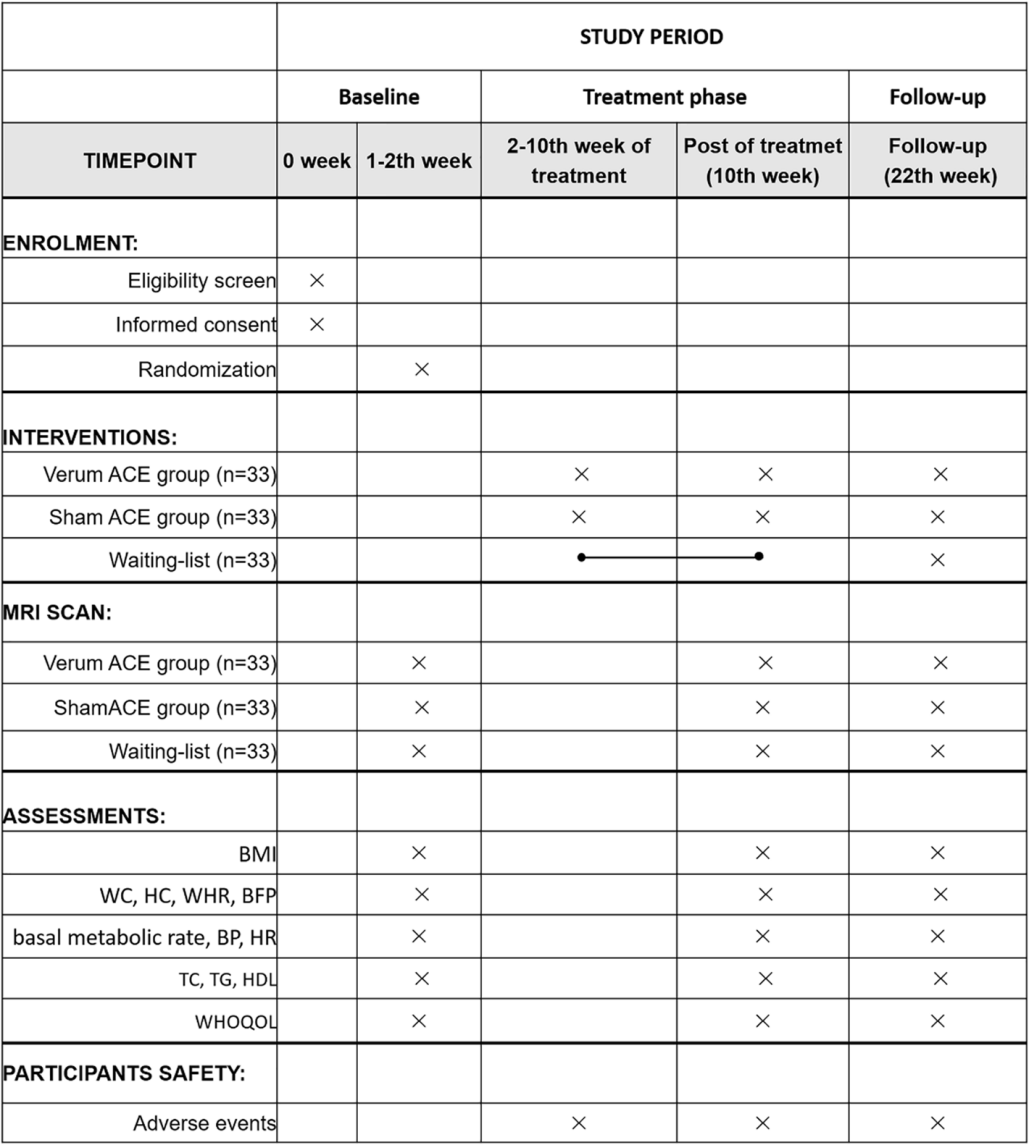
Study schedule for data collection

Patients who meet the inclusion criteria will be mainly recruited through outpatient clinics, online or offline advertisements (e.g., newspaper, poster, websites), or the WeChat public account of Shenzhen Traditional Chinese Medicine Hospital. If a patient is interested in joining the study, he/she can contact and consult with one of the researchers. Those patients who meet the inclusion criteria will be involved in the study. All the participants will sign a written informed consent form prior to the start of the study. The schedule of patient enrolment, intervention, and assessment is illustrated in Fig. 2.

### Inclusion criteria

Inclusion criteria are as follows:

1. Diagnosed with obesity according to Asian adult BMI criteria defined and proposed by the WHO Western Pacific region obesity working group in 2000
2. WC of ≥ 90 cm for males or ≥ 80 cm for females
3. Aged 18 to 65 years old
4. Does not have any taboos regarding catgut embedding therapy
5. Completes written informed consent

### Exclusion criteria

Patients with any one of the following criteria are excluded from this trial:

1. Endocrine disease (thyroid disease, pituitary disease, and diabetes mellitus) and autoimmune disease (systemic lupus erythematosus, Sjogren’s syndrome, and rheumatoid arthritis)
2. Metabolic disease such as hypertension and dyslipidemia
3. Other methods being used to control body mass and abdominal circumference, such as surgery, drugs, etc.
4. Women in pregnancy, nursing, perimenopause
5. Some severe diseases of the heart, liver, and kidney, or has tumor
6. Diagnosed with a psychiatric disorder
7. Participated in another study within 3 months

### Dropout criteria

1. Patients who do not meet the inclusion criteria but are mistakenly enrolled
2. Occurrence of severe AEs or complications which result in stopping the trial

### Sample size

The sample size has been calculated on the basis of results from a previous clinical trial [19]. The primary outcome in the present study is BMI reduction from baseline following 2 months of treatment. According to a previous study [19], in the ACE group, the BMI decreased 1.65 (standard deviation (SD) 1.24); in the sham ACE group, the BMI reduced 0.38 (SD 1.51). Considering a two-sided significance level of 0.05 and power of 0.90, 26 participants in each group are required as calculated by *t* test in G*Power software (version 3, Institute for Experimental Psychology, Heinrich-Heine-University, Germany). A dropout rate of 15% was taken into account so a total of 99 participants will be recruited in this trial (*n* = 33 participants in each group).

### Randomization and allocation concealment

If the participants meet all the inclusion criteria and sign the written informed consent form, they accept the principle of random allocation. Hence, 99 eligible participants will be randomly assigned to the verum ACE group, sham ACE group, and waiting list (WL) group at a ratio of 1:1:1. A random allocation sequence number will be generated using Statistical Analysis System software (SAS, version 9.1.3, SAS Institute Inc., Cary, NC, USA) by an independent statistician who will not be involved in the treatment or data collection. The sequential numbers are written on cards and sealed in an opaque envelope by an independent research assistant. After a participant randomly selects an opaque envelope and obtains an allocation sequence number, a research assistant will assign an identification code to them and record them in a case report form (CRF). Then, the result of a patient’s allocation will be given to the acupuncturists.

### Blinding

The acupuncturists are not blinded for the entire process. To eliminate potential bias, outcome assessors and statisticians are blinded to group assignment. Patient allocation will only be revealed under some special conditions, such as severe allergy, serious infection, uncontrolled pain, etc.

### Interventions

The ACE intervention is compliant with the Standards for Reporting Interventions in Clinical Trials of Acupuncture (STRICTA) guidelines. Patients in the verum ACE group, sham ACE group, and WL group will receive real ACE therapy, sham ACE therapy, and delayed active ACE therapy 20 weeks later, respectively. The course for the two ACE groups will comprise eight sessions over 8 weeks (one session per week).

### Verum ACE group

#### Acupoints

ACE treatment is a semi-standard method in this study. According to clinical practice, the literature, and the basic theory of traditional Chinese medicine, the prescription of ACE includes six obligatory acupoints and two groups of adjunct points.

**Fig. 3:**
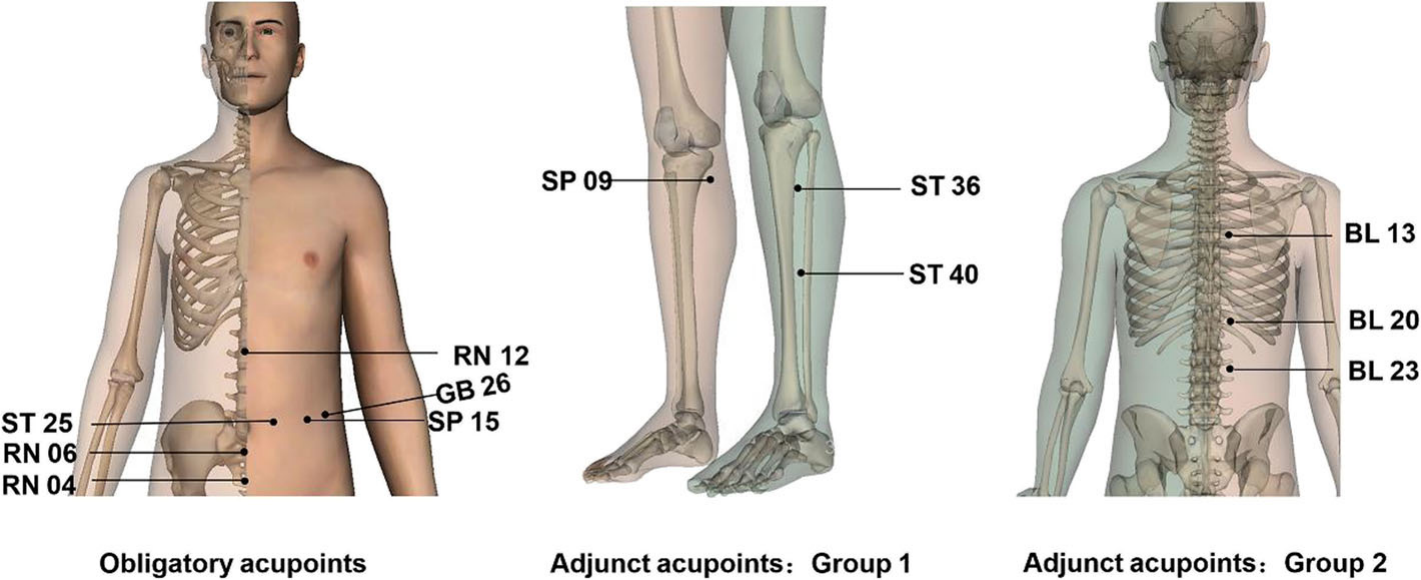
Locations of acupoints

The obligatory acupoints will include RN 12 (Zhongwan), ST 25 (Tianshu), SP 15 (Daheng), RN 06 (Qihai), RN 04 (Guanyuan), and GB 26 (Daimai). Adjunct acupoints will consist of two groups: acupoints of group 1 involve ST 36 (Zusanli), SP 09 (Yinlingquan), and ST 40 (Fenglong); acupoints of group 2 include BL13 (Feishu), BL20 (Pishu), and BL23 (Shenshu). The obligatory acupoints will be chosen in each session, and the adjunct points will be selected every other session. All acupoints will be localized according to the names and locations of acupoints drafted in 2006 by the National Standard of the People’s Republic of China (GB/T 12346–2006). Locations of the acupoints are presented in Table 1 and Fig. 3.

**Table 1:** Locations of acupoints in the two ACE groups

Acupoints	Locations
RN 12 (Zhongwan)	On the anterior median line, 4 cun above the umbilicus
ST 25 (Tianshu)	Level with the umbilicus, and 2 cun lateral to the anterior median line
SP 15 (Daheng)	Level with the umbilicus, and 4 cun lateral to the anterior median line
RN 06 (Qihai)	On the anterior median line, 1.5 cun below the umbilicus
RN 04 (Guanyuan)	On the anterior median line, 3 cun below the umbilicus
GB 26 (Daimai)	On the lateral abdomen, at the intersection of the vertical line of the free end of the 11th rib and the horizontal line on the same level of umbilicus, or 1.8 inch below LR 13 (Zhangmen) at the liver meridian
ST 36 (Zusanli)	3 cun directly below ST 35 (Dubi), and 1 digit lateral to the anterior margin of the tibia
SP 09 (Yinlingquan)	On the medial side of the calf, in the sunken spot between the medial lower margin of the tibia and the medial margin of the tibia
ST 40 (Fenglong)	8 cun directly below ST 35 (Dubi), and 2 digits lateral to the anterior margin of the tibia
BL13 (Feishu)	On the back, on the same level of the third subspinous of thoracic spine, and 1.5 cun lateral to the posterior midline
BL20 (Pishu)	On the back, on the same level of the 11th subspinous of thoracic spine, and 1.5 cun lateral to the posterior midline
BL23 (Shenshu)	On the back, on the same level of the second subspinous of lumbar vertebra, and 1.5 cun lateral to the posterior midline

#### Preparation of ACE

Catgut (Suzhou medical Co. Ltd, Jiangsu, China) of length 1.5 cm will be placed in front of an embedded needle (Zhengjianggaoguan medicine Co., Ltd., Shenzhen, China) for the operation. ACE will be performed under sterile conditions.

#### Operation procedures of ACE

Firstly, the acupuncturist should disinfect their hands with 75% alcohol and don medical gloves, medical mask, and medical hat. Simultaneously, patients will lie on the bed, supine or in a prone position according to the location of the acupoints, and will fully expose the skin of the acupoint area. Secondly, the acupoint regions will be sterilized with iodophor and alcohol twice by the acupuncturist. Simultaneously, an assistant will place the catgut in front of an embedded needle. Thirdly, the acupuncturist will locate the acupoint and then embed the needle into the skin at the appropriate place; consequently, the catgut will be embedded into the acupoint at a depth of about 1.5, 1, or 1 cm at the abdomen, lower limbs, and back, respectively. The embedded needle will be withdrawn once the patient has a feeling of soreness (*Deqi*); the catgut will be left under the tissue. Following this, sterile cotton balls will be pressed onto the acupoint for hemostasis and band-aids will be applied to the acupoints to prevent wound infection. Patients will be warned to avoid getting water on the embedded acupoints for 24 h.

The frequency of the ACE treatment will be once per week, lasting a total of 8 weeks.

### Sham ACE group

Patients in the sham ACE group will undergo a similar procedure to the verum ACE group except that nothing will be put into the catgut embedding needles before operation; thus, no catgut will be left under the patients’ acupoint tissue after needle extraction. The prescription of acupuncture will also be the same as that in the verum ACE group.

In the current study, all patients will be treated separately to avoid influencing each other. Both groups will be treated under the same conditions by acupuncturists, who will receive special training before participation in the study. During the entire ACE operation process, the acupuncturists and assistants will not talk about details.

### WL group

The WLC group will not have any intervention. The patients will be asked to receive delayed ACE therapy for free after a waiting period of 20 weeks.

### Magnetic resonance imaging data acquisition

In this study, magnetic resonance imaging (MRI) images of subcutaneous adipose tissue and visceral adipose tissue will be acquired by a MRI scanner (MAGNETOM; Siemens, Munich, Germany) with a matrix body coil of 18 channels at the MRI Center of Shenzhen Traditional Chinese Medicine Hospital. Before scanning, the participants will be trained in deep-breathing exercises. During the scanning process, they will be asked to hold their breath for about 15 s. The sequence parameters are as follows: flip angle, 65°; repetition time (TR)/echo time (TE), 195/3.69 ms; number of excitation (NEX), 1; matrix, 256 × 131; slice thickness, 7 mm; and echo train length, 4. In order to guarantee the passage of the image plane through the center of the vertebral disc between L4 and L5, the MRI data will be acquired from a sagittal scout. Qualitative image analysis will be performed by two independent reviewers as well.

### Outcome measurement

The clinical outcomes will be used to assess patients’ obesity levels and their quality of life. All measurements will be undertaken at baseline (after 8 weeks of treatments, and at 12 weeks of follow-up).

The primary outcome is the change of BMI from baseline. The BMI is calculated as follows: BMI = mass (kg)/(height(m))^2^. According to the BMI-based criteria presented by the WHO and Asia-Pacific classifications in 2000, BMI scores are ranked as “23.0–24.9 (pre-obesity),” “25–29.9 (level I obesity)”, and “more than 30 (level II obesity)”.

The secondary outcomes will include WC, hip circumference (HC), waist:hip ratio (WHR), body fat percentage (BFP), and the WHO Quality of Life (WHOQOL). WC will be measured by a stretch-resistant tape at the midpoint between the top of the iliac crest and the lower margin of the least palpable rib. In addition, HC will be measured around the widest portion of the buttocks using a tape parallel to the floor [23]. BFP will be measured with bioelectrical impedance.

WHOQOL is a widely used questionnaire for measuring physical and mental health status. The WHOQOL scale includes 26 items, involving four domains of patient quality of life: physical, psychological, social, and environmental [24, 25]. The total score ranges from 0 to 100. The lower the score, the poorer the patient’s quality of life.

Other outcome parameters, including basal metabolic rate, blood pressure (BP), heart rate (HR), total cholesterol (TC), triglyceride (TG), and high-density lipoprotein (HDL) levels, will be tested at each time-point.

### Statistical analysis

The statistical analysis will be conducted by independent statisticians who will be blinded to group allocation and intervention methods. Before data analysis, the research group will draw up a statistical plan, including the required data and method of data processing.

Data will be analyzed using SPSS 22.0 software (IBM, Armonk, NY, USA). For MRI data, the images will be analyzed on a workstation (Syngo Multimodality Workplace) for the quantification of VAT and SAT.

Demographic information and levels of measured variables will be analyzed by descriptive statistics. Categorical data are described as percentage (n%) or analyzed using the Chi-square (x^2^) test. Additionally, for continuous variables, if data are normally distributed, one-way analysis of variance (ANOVA) will be used to detect differences among the three groups. Otherwise, the Kruskal–Wallis (K-W) test can be considered. The longitudinal and repeated measured data will be analyzed by the repeated measure analysis.

In this study, SFA is quantified as an area between the outline of the abdominal skin and the outer abdominal muscle, while VFA is defined as the enterocoelia and retroperitoneal region between the inside edge of the abdominal muscles and the spinal front. Detailed methods have been described previously [26–28]. The correlation coefficients between two reviewers who analyzed the same image for SFA and VFA (*n* = 30) were r = 0.99, *P* < 0.001 and r = 0.98, P < 0.001, respectively.

Eventually, the Pearson’s correlation between the changes of SFA and VFA and improvement of clinical variables will be calculated in each group.

For the above-mentioned statistical analyses, a *P* value < 0.05 is considered statistically significant.

### Safety

To guarantee that the ACE operation is standard and safe, the acupuncturists in this study should pay attention to the following: (1) ensure the operation is strictly aseptic to prevent infection; (2) catgut should not be embedded in adipose tissue that could prevent fat liquefaction; (3) catgut should not be exposed to the body surface to prevent infection; (4) master the angle and depth of the embedding in order to avoid injury to the internal organs, great vessels, nerves, etc.; (5) acupoints can’t be wet for 24 h after embedding; (6) inform patients that they may experience soreness, distension, and numbness at the acupoints for one or two days, and possibly more than three to five days.

### Management of adverse events

ACE therapy may lead to different AEs, such as fainting during operation, subcutaneous hematoma, allergy, infection, severe pain, etc. Any AEs experienced by participants should be reported to the researchers. After confirming the validity of the AEs by an evaluator, the acupuncturists will immediately stop the treatment procedure and deal with the AEs. All AEs, as well as management of the AEs, will be carefully recorded during treatment and follow-up phases.

### Quality control

Acupuncturists, assistants, data collectors, and statisticians who participated in the study should abide by the rules and regulations. Before the study, each researcher took basic study training to understand the design, purpose, and basic information about this research. The acupuncturists should have at least 3 years of practical acupuncture experience, and they also should be familiar with the operation process and be able to cope with any possible AEs. Data collectors are responsible for saving and managing various data, and strictly proofread data. Patient withdrawals and AEs during the study will be recorded in detail. Statisticians will be fully responsible for data management and statistical analysis. Regular team meetings will be held and fully documented.

### Ethics approval

This study was approved by the Chinese Clinical Trial Registry. The registration number of this trial is ChiCTR1800020248. In addition, the study protocol was approved by the Ethics Committee of Shenzhen Traditional Chinese Medicine Hospital. Prior to start of the study, patients will be informed about the potential risks of the study. Patients voluntarily participate in the study with informed consent. If the protocol needs to be amended, all the materials on trial will be reported to the Ethics Committee, and the amended protocol can only be implemented after consent is acquired.

### Data management and monitoring

The research associates will record the information on the CRFs and verify that the data is fully, swiftly, and accurately collected. The private information and medical records of patients, including their name, phone number, and ID number, will be anonymous to ensure confidentiality. All the research documents will be kept in specialized cabinets and preserved for at least 5 years after publication.

In addition, a data monitoring committee is established consisting of experienced experts in the Good Clinical Practice Department of the Shenzhen Traditional Chinese Medicine Hospital to periodically review the progress of the trial and monitor collection of the data, allocation concealment, etc. The modification or termination of the trial can be performed by the committee. The data monitoring committee is independent from the sponsor and has no conflict of interest.

## Discussion

It is noteworthy that ACE is extensively applied for weight loss. Although previous studies have shown that ACE might be effective in improving overweight, high-quality trials with rigorous design are still urgently required to assess the effects of ACE on obesity [29]. The results of the present study may contribute to a better understanding of how ACE exerts its potential therapeutic effects on obesity.

Recently, increasing attention has been paid to the visceral fat of obesity, which is related to energy storage, and is considered as an endocrine and paracrine organ, influencing a number of metabolic processes by releasing cytokines and bioactive mediators [30–33]. Body weight can be adjusted by cytokines and mediators. Thus, VFA is a significant indicator for estimating metabolic risk in obese populations [34, 35]. In previous studies, however, the influence of ACE on obesity was mainly assessed by BMI or WC, and a limited number of studies have assessed those influences in relation to VFA. Hence, evaluation in this study is done according to both standard obesity-related indices (BMI, WC, etc.) and VFA in order to clearly illuminate the effects of ACE on obesity.

The purpose of this study is to compare measurement changes after 2 months of treatment with three different intervention methods: verum ACE, sham ACE, and no intervention (WLC group). Several studies [36, 37] have indicated that a sham-controlled design could separate specific and non-specific effects, which may play a pivotal role in evaluating the effectiveness of treatment. Thus, the ACE and sham ACE treatments are both applied to investigate the anti-obesity effects in this study. The sham ACE treatment involves only needle piercing into the acupoints without catgut being fixed; the rest of the procedure is the same as in the ACE group. Thus, it can successfully blind patients and estimators; as a result, it can minimize the placebo effect. In addition, as far as non-specific effects (sham ACE versus no intervention) and bias caused by psychological influences, the third group without any intervention is considered to avoid the placebo effects of group allocation or patients’ beliefs on weight loss. A possible limitation of this trial is that compliance might be difficult for participants due to the long interval from the end of the 2 months of treatment to the follow-up at 3 months. Several actions, such as phone interview, should be taken to improve compliance.

The design and methodological rigor of this trial may hopefully provide consolidated evidence regarding the efficacy and safety of ACE for treating obesity, through collecting valuable and high-quality data, and also contribute to future research in ACE therapy.

## Trial status

This trial is currently recruiting patients. The trial began recruitment on January 1, 2019 and is anticipated to be completed on December 31, 2021. The version number and date of the protocol are v1.0, and October 25, 2018, respectively.

## Supplementary information

**Additional file 1.** SPIRIT 2013 checklist.

## Data Availability

The full data from this study will be available upon reasonable request after completion of the study.

## Abbreviations

ACC: American College of Cardiology
ACE: Acupoint catgut embedding
AE: Adverse event
BFP: Body fat percentage
BMI: Body mass index
BP: Blood pressure
CVD: Cardiovascular disease
FDA: Food and Drug Administration
GHO: Global Health Observatory
HC: Hip circumference
HDL: High-density lipoprotein
HR: Heart rate
MRI: Magnetic resonance imaging
NHLBI: National Heart, Lung and Blood Institute
NIH: National Institutes of Health
RCT: Randomized controlled trial
SD: Standard deviation
SFA: Subcutaneous fat area
STRICTA: Standards for Reporting Interventions in Clinical Trials of Acupuncture
T2D: Type 2 diabetes
TC: Total cholesterol
TG: Triglyceride
VFA: Visceral fat area
WC: Waist circumference
WHO: World Health Organization
WHOQOL: World Health Organization Quality of Life
WHR: Waist:hip ratio
WT: Waiting list
